# A comprehensive non-uniformity correction method for EMCCD

**DOI:** 10.1038/s41598-021-03478-3

**Published:** 2021-12-14

**Authors:** Li Qiao, Mingfu Wang, Zheng Jin, Danbo Mao

**Affiliations:** 1 Institute of Optics and Electronics, Chinese Academy of Sciences, Chengdu, 610200 Sichuan China; 2 Key Laboratory of Science and Technology on Space Optoelectronic Precision Measurement, Chinese Academy of Sciences, Chengdu, China

**Keywords:** Electrical and electronic engineering, Nonlinear phenomena, Statistical physics, Computational science, Statistics, Photonic devices, Electronics, photonics and device physics, Techniques and instrumentation

## Abstract

The non-uniformity of image directly affects the application of EMCCD in various disciplines. The proposed method can significantly improve the uniformity of EMCCD output image. The correction algorithm of "reverse split and forward recovery" is determined through analyzing the imaging model of EMCCD, and the comprehensive non-uniformity correction function model is established. The 8-tap EMCCD chip CCD220 of British e2v company is used for experimental verification. The results show that after the comprehensive correction the consistencies of the light response characteristic curve and the multiplication gain curve of each channel in EMCCD are obviously improved, and also the photo response non-uniformity (PRNU) of the output image is substantially reduced from 24.5 to 4.1%, which prove the effectiveness of the proposed method.

## Introduction

EMCCD is a typical photo-detector used in weak light environment, of which the internal imaging process mainly includes photoelectric conversion, charge transfer, multiplication amplification, and A/D conversion. The charge is horizontally transferred and serially sent to the multiplication register for multistage amplification to achieve desired exponential multiplication in the process of multiplication^1^, which is the key step for EMCCD to image under weak light signal and it has been widely used in fields such as astronomical observation, biomedicine, quantum science and so on^2–10^.

However, the uniformity is the key index to evaluate the quality of output image of EMCCD, which directly affects the application of EMCCD in various disciplines. There are many evaluation methods of non-uniformity reported^11–16^. In this paper, the photo response non-uniformity (PRNU) of image sensor is used to evaluate the uniformity of EMCCD output image. Compared with the common CCD, EMCCD adds a multiplication register between the readout register and the readout amplifier. The camera works in the normal CCD mode when the multiplication register is off, while it works in EMCCD mode the multiplication register amplifies the charge exponentially. In order to improve the maximum output frame rate, EMCCD often adopts multi-channel output configuration, which will directly affect the uniformity of EMCCD output image. The non-uniformity in EMCCD is the result of the interference between the non-uniformity in channel and that between channels. The research on the non-uniformity correction of ordinary CCD imaging mainly includes single point method, two point method, multipoint fitting method, self-adaptive method^17–23^, etc. Because of the different imaging principle, the non-uniformity correction method of ordinary CCD is not suitable for EMCCD. The non-uniformity correction of EMCCD includes not only the non-uniformity correction of imaging model of ordinary CCD, but also the non-uniformity correction of real multiplication gain^24,25^. The aforementioned methods only focus on the single component of non-uniformity in the imaging process, there is no relevant research on the comprehensive non-uniformity correction of EMCCD.

In this paper, a method of comprehensive non-uniformity correction of EMCCD is proposed, which fully considers the relationship between the non-uniformity in channel and that between channels. The method of "reverse split and forward recovery" is adopted to realize the comprehensive correction of non-uniformity for EMCCD imaging process, after which the quality of EMCCD output image greatly improves, and it provides a theoretical basis for its wider application in scientific or commercial research in weak light environment.

## Non-uniformity in EMCCD

**Figure 1:**
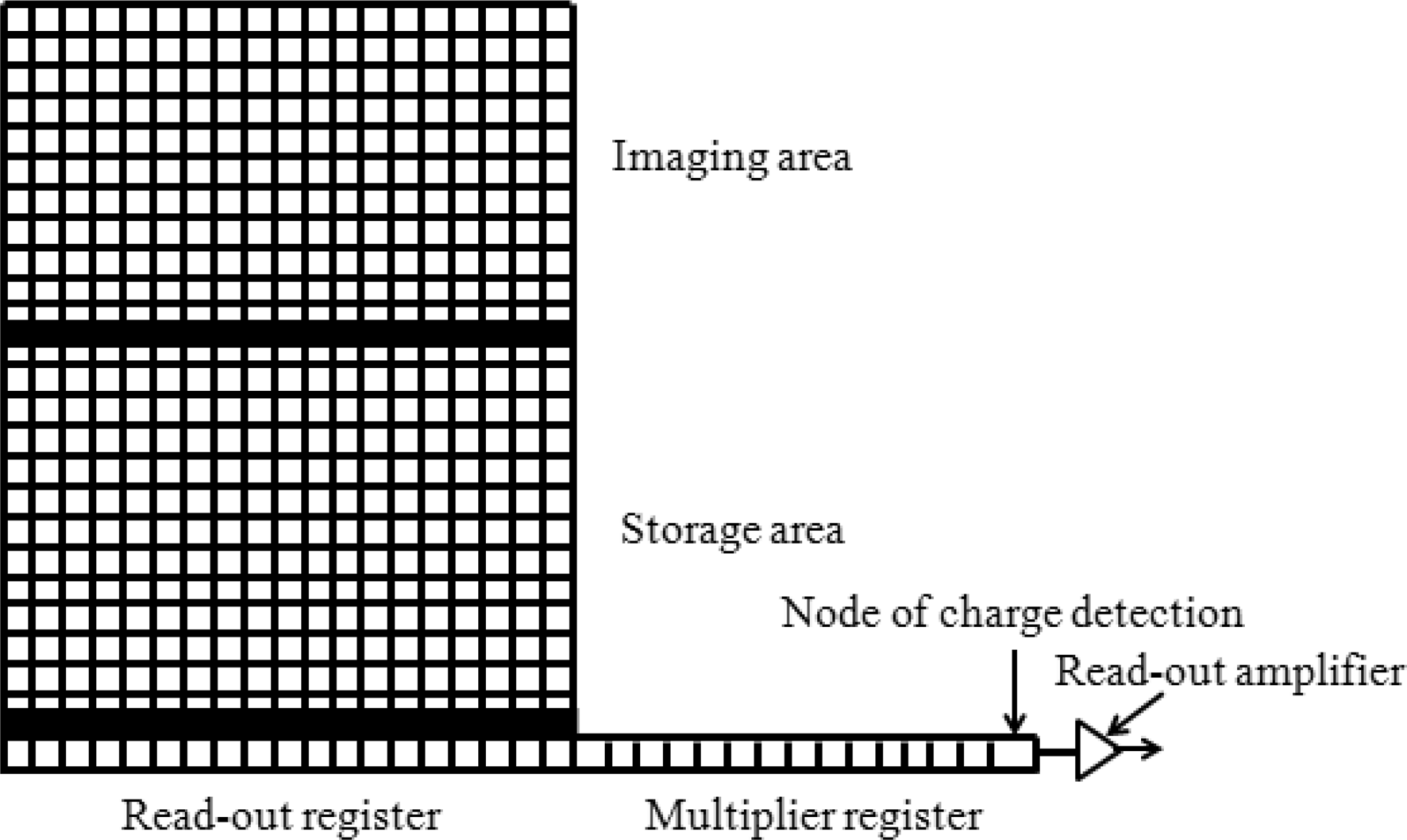
Internal working principle of EMCCD.

The imaging process of EMCCD is shown in Fig. 1. The photo electric conversion takes place in the imaging area under the illumination of light to engender photo generated charges, which are driven by the internal clock and transferred to the storage area, where the charges are vertically transferred to the readout register and then horizontally transferred to the multiplication register, in which the photo generated charges are exponentially amplified and sent to the readout amplifier and the back-end circuit for processing, and finally the image will be output^26^. Obviously, the non-uniformity in EMCCD is introduced in the above process.

### Non-uniformity composition in EMCCD

According to the source of introduction, the non-uniformity in EMCCD is broadly categorized into two types: the non-uniformity between pixels and the one between channels.

1. The non-uniformity between pixels

When EMCCD works in the normal CCD mode, the non-uniformity introduced during the camera working process mainly includes that of the photoelectric conversion in the imaging area, that introduced in the charge transfer process, that of the readout amplifier and the back-end circuit and the background signal. As the source of the introduction of the above-mentioned non-uniformities is a single pixel, they can be defined as the non-uniformities between pixels.

(2) The non-uniformity between channels

The calculation of true multiplication gain of photo-generated charge in the multiplication register is shown in Eq. (1) ^1^.

1 $$G={\left(1+r\right)}^{N}$$

In this equation, *N* is the number of stages in multiplication register; *r* is the additional multiplication factor, which is positively correlated to the multiplication voltage $\Delta v$; *G* is the true multiplication gain.

It can be seen from Eq. (1) that the true multiplication gain *G* is directly related to the additional multiplication factor $r$ of the single-level multiplication register, that is, it is directly related to the multiplication voltage $\Delta v.$ Because EMCCD adopts multi-tap output structure, the circuit difference between channels will bring in the inconsistency of multiplication voltage $\Delta v$ incurring the non-uniformity between channels. Therefore, the gain non-uniformity of each channel in the multiplication register can be defined as the non-uniformity between channels.

### The logic relationship of introduced non-uniformity

**Figure 2:**
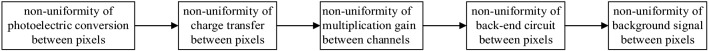
The logic relation of introduced non-uniformity in EMCCD.

The logic relationship of introduced non-uniformity in EMCCD is shown in Fig. 2. The first photo generated charge is inconsistent due to the difference of photoelectric conversion efficiency between pixels when the uniform light irradiates the imaging area of EMCCD, which leads to the non-uniformity of photoelectric conversion. There also exits the difference between pixels during the vertical and horizontal transfer, which brings in the non-uniformity of charge transfer. The inconsistency of the multiplication voltage $\Delta v$ due to the difference of the circuit of each channel of EMCCD will lead to the non-uniformity of the multiplication gain after the charge is sent into the multiplication register. The non-uniformity of the back-end circuit is also introduced after the charge is sent to the back-end circuit for conversion and output. Finally, the signal of the back-end circuit and the background are superimposed to form the final output image, which draws in the non-uniformity of the background signal.

## The principle of the comprehensive non-uniformity correction of EMCCD

### Imaging model of EMCCD

The imaging model of single pixel in EMCCD is shown in Eq. (2).

2 $${image}_{out}={k}_{1}*\left[\left({k}_{0}*photons+{b}_{0}\right)*G\right]+{b}_{1}+{image}_{dark}$$

In this equation, $photons$ is the original signal of a single pixel entering the imaging area; ${k}_{0}$ and ${b}_{0}$ are the comprehensive linear photoelectric conversion coefficients before the photo generated charge of a single pixel enters the multiplication register;$G$ is the true multiplication gain of the charge entering the channel of the current pixel; ${k}_{1}$ and ${b}_{1}$ are the comprehensive linear photoelectric conversion coefficients of the charge amplified by the multiplication register in the back-end circuit; ${image}_{dark}$ is the background signal superimposed with the charge.

Coefficient ${b}_{0}$ in Eq. (2) is related to the dark current introduced during charge transfer. The exposure time is usually controlled at millisecond level when EMCCD works normally, so the effect of dark current can be ignored. Therefore, Eq. (2) can be simplified as follows:

3 $${image}_{out}={k}_{1}*\left[{k}_{0}*photons*G\right]+{b}_{1}+{image}_{dark}$$

After expanding Eq. (3), we get the following results:

4 $${image}_{out}=k*photons*G+b+{image}_{dark}$$

In this equation,$k$ and $b$ are the comprehensive linear photoelectric conversion coefficients of a single pixel in the whole EMCCD imaging process.

Equation (4) describes the imaging model of a single pixel in EMCCD. The $k$, $b$ and ${image}_{dark}$ vary with pixels, and *G* varies with channels.

5 $${image}_{out}(i,j)=k(i,j)*photons*G(i,j)+b(i,j)+{image}_{dark}(i,j)$$

Equation (5) represents the imaging model of all pixels in EMCCD.

In this equation, $i$ and $j$ are the i-th row and j-th column of the EMCCD output image, respectively;$G\left(i,j\right)$ is the true multiplication gain of the channel of the current pixel.

### Principle of comprehensive non-uniformity correction

As is shown in Eq. (5), the difference of the comprehensive linear photoelectric conversion coefficients $k(i,j)$ , $b(i,j)$ and the background signal ${image}_{dark}(i,j)$ between pixels and that of the real multiplication gain $G\left(i,j\right)$ among channels will lead to the non-uniformity of the final EMCCD output image when the original signal $photons$ are consistent. Therefore, the method of reverse split and forward recovery is adopted to correct the non-uniformity of the whole imaging process of EMCCD.

#### Coefficient computation

(a) Make EMCCD work in the normal CCD mode and turn off the original signal $photons$ to obtain multiple background images, which is the background signal ${image}_{dark}(i,j)$ of the current camera.

(b) Make EMCCD work in the normal CCD mode and segmentally acquire the output image of the camera under different original signal $photons\left[{\mathrm{n}}\right]$(n ≥ 20). Take the original signal $photons$ as the X-axis and the difference of output image and background signal ${image}_{dark}\left(i,j\right)$ as the Y-axis for linear fitting to obtain the comprehensive photoelectric linear conversion coefficient $k(i,j)$ and $b\left(i,j\right)$ corresponding to each pixel.

(c) Make EMCCD work in the normal CCD mode to obtain the output image of the current camera when the multiplication register is free $(G=1$). The original signal ${photons}_{0}$ is reversely reckoned as ${P}_{0}$ using the obtained ${image}_{dark}\left(i,j\right), k\left(i,j\right) and b(i,j)$ according to Eq. (5).

(d) Make EMCCD work in the multiplication CCD mode and keep the original signal ${photons}_{0}$ unchanged to obtain the output image under different gain voltages $\Delta v[m]$($m$≥30). Similarly, the signal after multiplication ${P}_{1}$ could be reversely calculated to be ${photons}_{0}*G(i,j)$.

(e) The real multiplication gain under different voltages $\Delta v\left[m\right]$ (m ≥ 30) could be calculated by dividing ${P}_{0}$ into ${P}_{1}$. Take the multiplication voltages $\Delta v$ as the X-axis and the corresponding real multiplication gain $G(i,j)$ as the Y-axis for exponential fitting to infer the function relation between the multiplication gain $G(i,j)$ and the voltage $\Delta v$.

#### Reverse split

Record the current EMCCD operating voltage $\Delta {v}_{origin}$ to obtain an uncorrected original output image ${image}_{origin}$. The original signal corresponding to the current image can be reversely calculated according to Eq. (6).

6 $${photons}_{origin}(i,j)=\frac{{image}_{origin}\left(i,j\right)-{image}_{dark}\left(i,j\right)-b(i,j)}{k\left(i,j\right)*{G}_{i,j}(\Delta {v}_{origin})}$$

In this equation, ${G}_{i,j}(\Delta {v}_{origin})$ is the true multiplication gain after substituting the multiplication voltage $\Delta {v}_{origin}$ into ${G}_{i,j}(\Delta v)$; ${photons}_{origin}$ is the original signal corresponding to the original output image ${image}_{origin}$.

#### Forward recovery

The original signal obtained in section “Reverse split” is forward recovered according to Eq. (7) to gain an image with comprehensive non-uniformity correction.

7 $${image}_{modify}(i,j)={k}_{ave}*{photons}_{origin}(i,j)*{G}_{ave}+{b}_{ave}+{image}_{darkAve}$$

In this equation, ${k}_{ave}$ and ${b}_{ave}$ are the mean value of $k\left(i,j\right)$ and $b(i,j)$, respectively, in Eq. (6); ${image}_{darkAve}$ is the average of background signal ${image}_{dark}$; ${G}_{ave}$ is the average of the true multiplication gain of each channel of EMCCD under the current multiplication voltage $\Delta {v}_{origin}$.

#### Determination of comprehensive correction coefficient

The comprehensive correction equation of EMCCD is obtained by substituting Eq. (6) into Eq. (7) as follows:

8 $${image}_{modify}\left(i,j\right)={k}_{modify}\left(i,j\right)*{G}_{modify}\left(i,j\right)*\left[{image}_{origin}\left(i,j\right)-{b}_{modify0}\left(i,j\right)\right]+{b}_{modify1}$$

$${b}_{modify0}\left(i,j\right)={image}_{dark}\left(i,j\right)+b(i,j)$$

$${G}_{modify}(i,j)=\frac{{G}_{ave}}{{G}_{i,j}(\Delta {v}_{origin})}$$

$${k}_{modify}(i,j)=\frac{{k}_{ave}}{k\left(i,j\right)}$$

$${b}_{modify1}={b}_{ave}+{image}_{darkAve}$$

In this equation, ${G}_{modify}$ is the gain correction factor of the channel of the current pixel.

## Experimental and results

### Hardware device

The CCD220 chip of British e2v company possesses 8-tap output structure, which can improve the maximum output frame rate of EMCCD and is a typical chip used in weak light environment. The resolution of the final output image is 240 (row) × 240 (column), and it is composed of eight channels. CCD220 chip is taken as an example to verify the effectiveness of the proposed algorithm in this paper.

**Figure 3:**
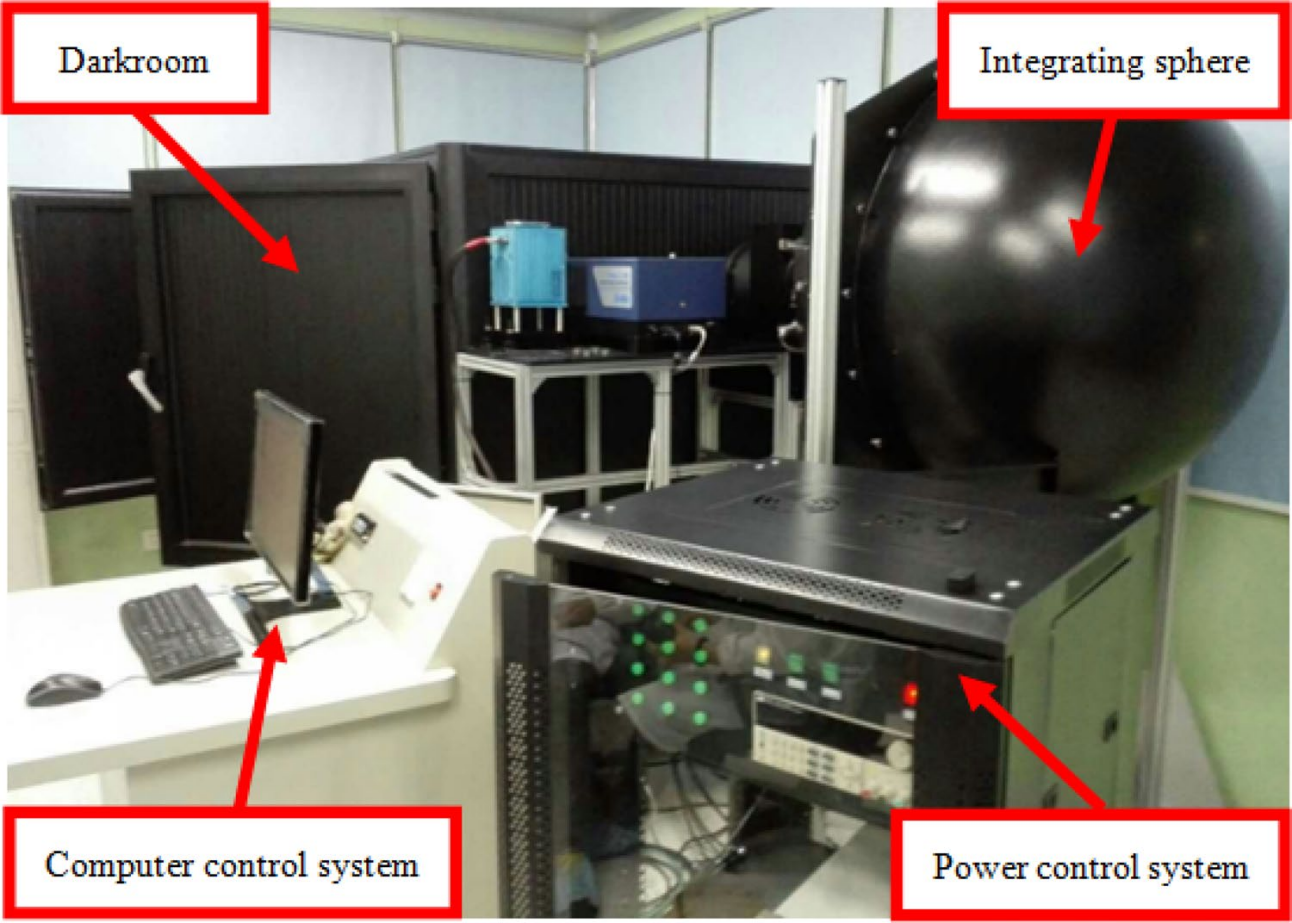
Photoelectric performance testing system of EMCCD.

Figure 3 shows the image acquisition system of this experiment, which is mainly composed of integrating sphere with the aperture of the light outlet about 20 cm and the brightness uniformity more than 97%, darkroom with built-in slide rail and the camera bracket mounted, power control system and computer control system. The EMCCD is installed on the bracket and slides with the rail. The distance *d* between the target surface of the camera and the light outlet of the integrating sphere is 1.2 m. The physical width *D* of CCD220 chip target surface is 5.76 mm, which conforms to the consistency criterion of light source^27^. The integrating sphere has four levels of transmittance of 100%, 10%, 1% and 0.1%, which can provide the weak light environment for EMCCD when it works in the multiplication gain mode.

### Experiment

This section describes the solution procedure of all correction parameters in detail based on the principle of comprehensive non-uniformity correction of EMCCD.

#### Calculation of correction coefficient of non-uniformity between pixels

The correction coefficients of non-uniformity between pixels includes those of the background signal ${image}_{dark}\left(i,j\right)$, the comprehensive photoelectric linear conversion coefficients $k\left(i,j\right)$, $b(i,j)$, ${k}_{ave}$ and ${b}_{ave}$.

1. Calculation of background signal correction coefficient

**Figure 4:**
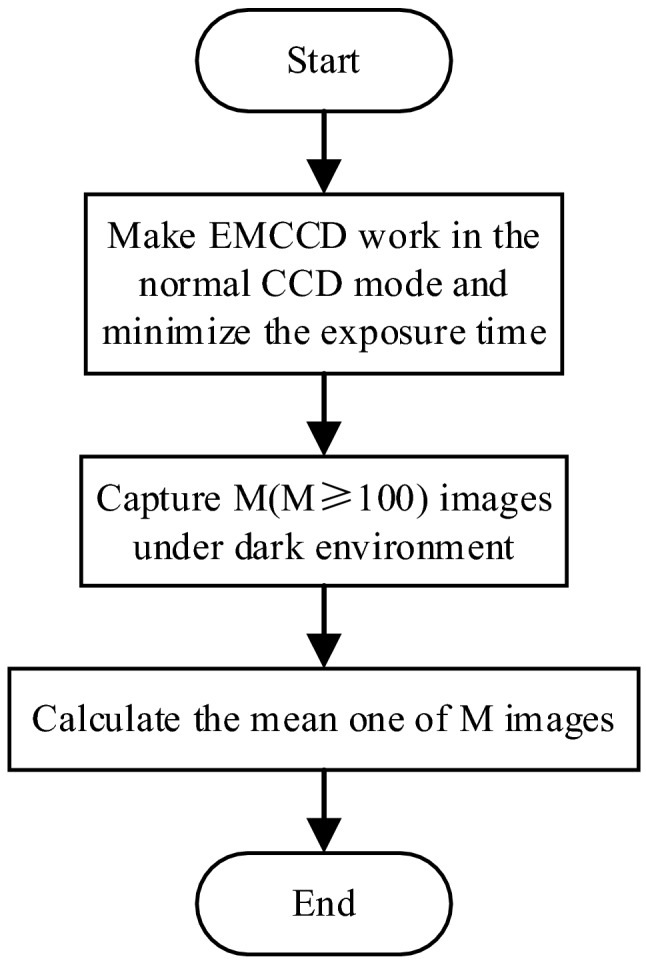
Calculation flow chart of background signal correction coefficient.

The mean value image in Fig. 4 is the correction coefficient of background signal ${image}_{dark}\left(i,j\right).$

(2) Calculation of comprehensive photoelectric linear conversion coefficients

**Figure 5:**
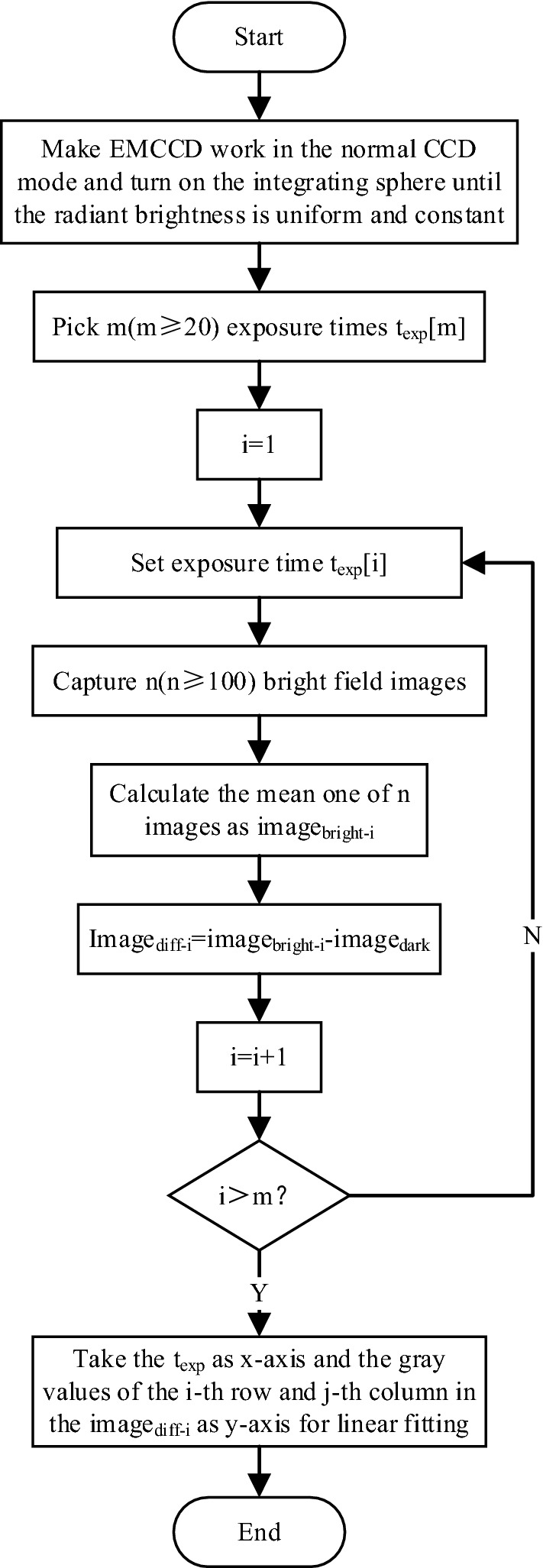
Calculation flow chart of comprehensive photoelectric linear conversion coefficient.

The fitting coefficients $k\left(i,j\right)$ and $b(i,j)$ in Fig. 5 are the comprehensive photoelectric linear conversion ones. ${k}_{ave}$ and ${b}_{ave}$ can be obtained from Eq. (9) as follows.

9 $${k}_{ave}=\frac{\sum_{i=1}^{row}\sum_{j=1}^{col}k(i,j)}{row*col} {b}_{ave}=\frac{\sum_{i=1}^{row}\sum_{j=1}^{col}b(i,j)}{row*col}$$

In this equation, $row$ and $col$ represent the number of rows and columns in a single image, respectively.

#### Calculation of correction coefficients of non-uniformity between channels

${G}_{i,j}(\Delta {v}_{origin})$ and ${G}_{ave}$ are the non-uniformity correction coefficients between channels.

**Figure 6:**
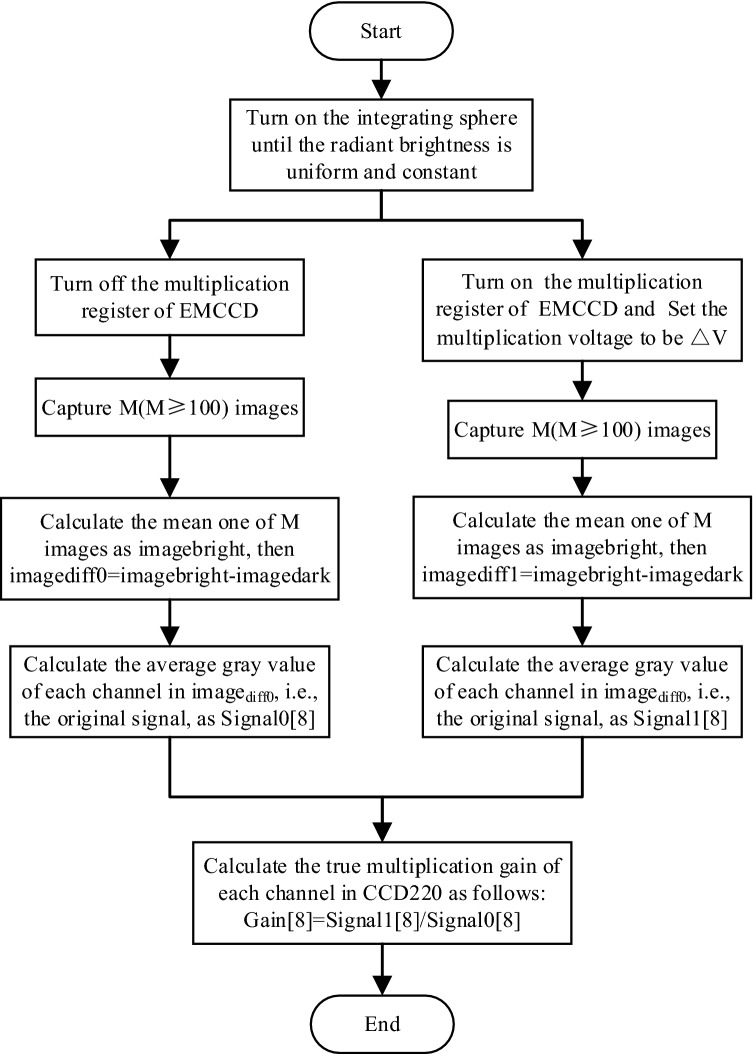
Calculation flow chart of true multiplication gain when gain voltage is $\Delta v$.

Figure 6 shows the calculation of the true multiplication gain of 8 channels in CCD220 when the gain voltage is $\Delta v$. The true multiplication gain of each channel can be obtained under m (m ≥ 30) different gain voltages. As for the first channel of CCD220, take the m gain voltages $\Delta v$ as the X-axis and the corresponding real multiplication gain $G$ as the Y-axis for exponential fitting to obtain the functional relationship between them. Also the respective function relation of the remaining seven channels can be determined through the same fitting method.

The current true multiplication gain of each channel ${G}_{i}(\Delta v)$ can be calculated by substituting the gain voltage $\Delta v$ into the function relation of each of the eight channels, the mean value ${G}_{ave}$ corresponding to the current gain voltage $\Delta v$ is calculated as follows:

10 $${G}_{ave}=\frac{\sum_{i=1}^{8}{G}_{i}(\Delta v)}{8}$$

The function prototype for a single channel is shown in Eq. (11) and the coefficients of fitting functions of each channel in CCD220 are shown in Table 1.

**Table 1:** Coefficients of fitting functions of each channel in CCD220.

Coefficients	Channel 1	Channel 2	Channel 3	Channel 4	Channel 5	Channel 6	Channel 7	Channel 8
a (e^−14^)	8.5009	8.5264	9.4042	8.8456	7.8113	5.0044	7.3359	7.9563
b	8.7199	8.7220	8.6911	8.7060	8.7524	8.8741	8.7621	8.7372

11 $$G={e}^{(a*{\Delta v}^{b})}$$

### Experimental result

1. Verification of light response characteristics

**Figure 7:**
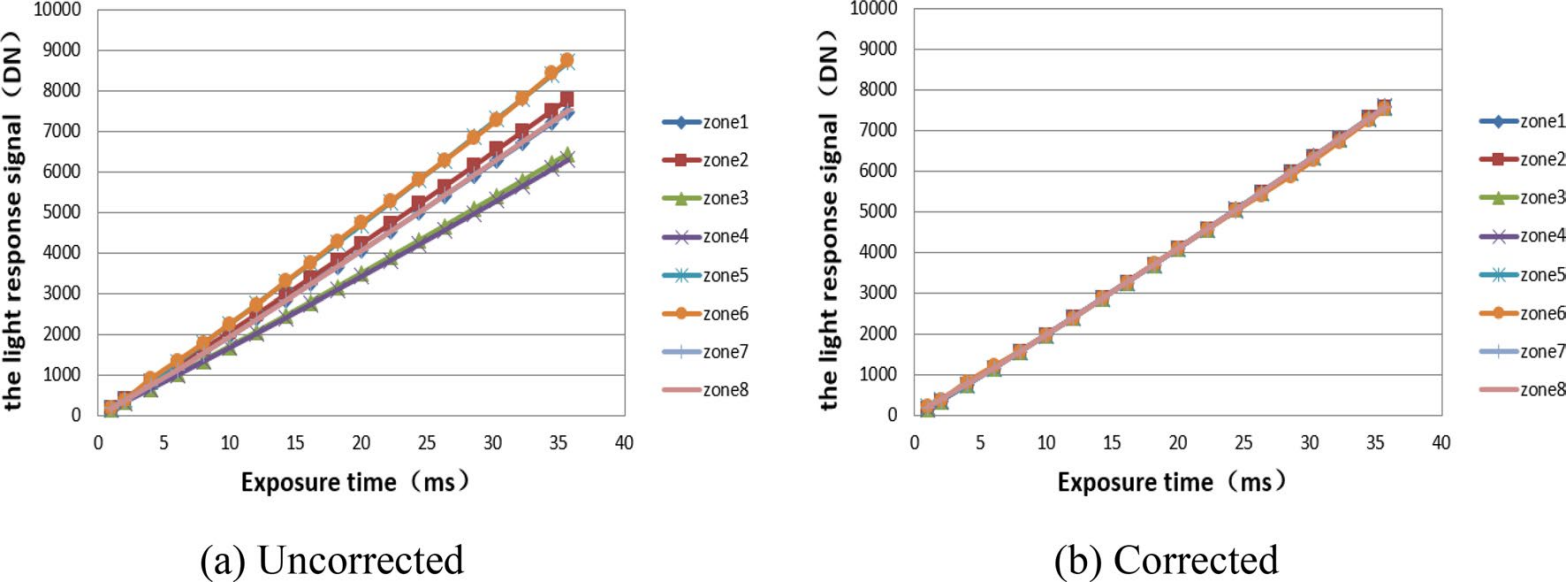
Light response curves of EMCCD before and after comprehensive non-uniformity correction.

Figure 7 shows the light response curves of the output images of 8 channels before and after correction when EMCCD works in the normal CCD mode. The light response signals of the 8 channels increase linearly with the extension of exposure time. As it is shown in Fig. 7(a) that the longer the exposure time is, the greater the difference of the light response signal of the eight channels is, and the worse the uniformity of the image is. However, the result is greatly improved as shown in Fig. 7(b) that the difference of light response signal between channels no longer increases with the exposure time, and the non-uniformity introduced is effectively suppressed.

(2) Verification of multiplication characteristics

**Figure 8:**
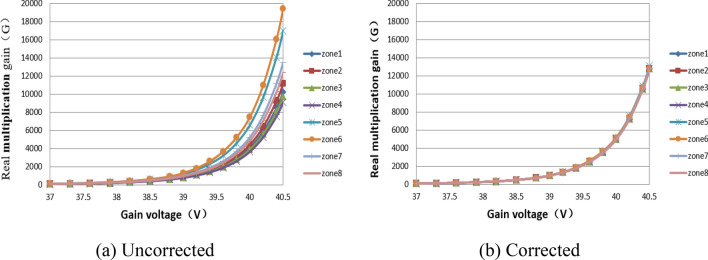
Comparison of true multiplication gain curves before and after correction.

Figure 8 shows the comparison of the true multiplication gain curves of the output images of the eight channels before and after correction when the EMCCD works in the multiplication mode. The real multiplication gains of the eight channels increase exponentially with the increase of gain voltage. As it is shown in Fig. 8(a) that the larger the gain voltage is, the greater the difference of the true multiplication gain of the eight channels is, and the worse the uniformity of the image is. In contrast, the result is greatly improved as shown in Fig. 8(b) that the difference of the true multiplication gain between channels no longer increases with the gain voltage. The non-uniformity of the image induced by the multiplication gain is effectively suppressed.

(3) Comparison of images

The Photo response non-uniformity, i.e., PRNU, of image sensor is a typical index for evaluating image non-uniformity^27^, of which the detailed calculation is shown in Eq. (12). The smaller the value of PRNU, the better the uniformity and quality of the image.

12 $${PRNU}_{1288}=\frac{\sqrt{{{s}_{bright}}^{2}-{{s}_{dark}}^{2}}}{{\mu }_{bright}-{\mu }_{dark}}$$

In this equation, ${\mu }_{bright}$ and ${s}_{bright}$ are the mean value and standard deviation of the whole image, respectively. ${\mu }_{dark}$ and ${s}_{dark}$ are the mean value and standard deviation of the corresponding dark field image, respectively.

**Figure 9:**
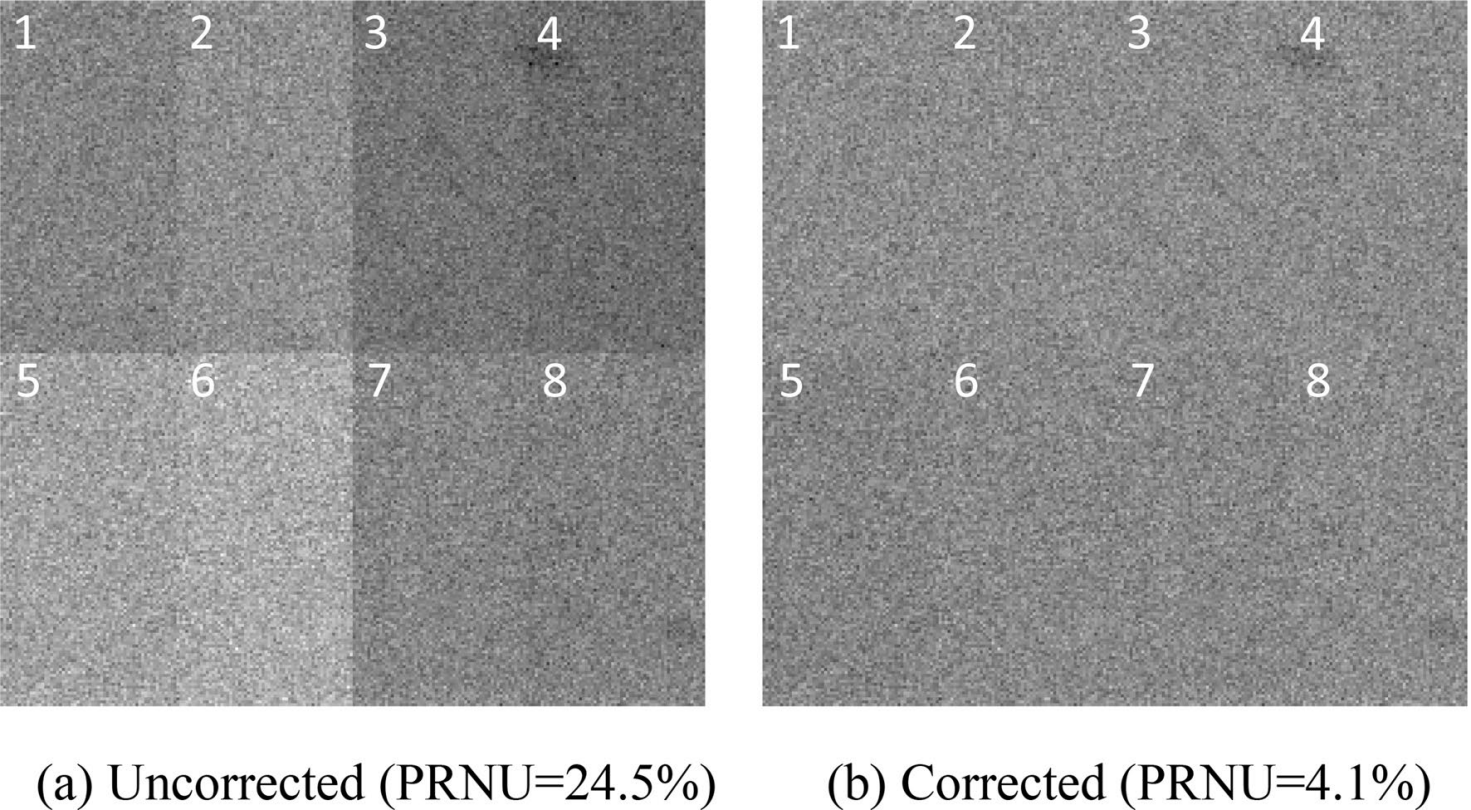
Image comparison before and after correction when the gain voltage is 40.0 V.

Figure 9 shows the original image output by EMCCD and the image after comprehensive non-uniformity correction when the gain voltage $\Delta v$ is 40 V in weak light environment. Because of the inherent multi tap physical characteristics of CCD220 chip, the original output image of EMCCD in Fig. 9(a) is divided into 8 channels with poor uniformity and PRNU being 24.5%. However, the uniformity of the image in Fig. 9(b) is significantly improved after the comprehensive correction, with the PRNU dramatically reduces to 4.1%.

## Conclusion

In this paper, a comprehensive correction method for non-uniformity of EMCCD is proposed. The non-uniformity is categorized into two groups: the one between pixels and that between channels. The logical relationship between the influencing factors of non-uniformity in EMCCD is determined after the imaging model is put forward by analyzing the imaging principle of EMCCD, and then the algorithm of "reverse split and forward recovery" is finally established. The 8-tap EMCCD chip CCD220 of e2v company is used for experimental verification. The results show that after the comprehensive correction of non-uniformity, the difference of light response between pixels no longer increases with the exposure time, the consistency of light response curves of each channel is significantly improved, and the introduced non-uniformity between pixels is effectively suppressed when CCD220 works in the ordinary CCD mode. The results also show that after the comprehensive correction, the difference of the multiplication gain between channels no longer increases with the gain voltage, the consistency of the multiplication gain curve of each channel is improved obviously, the introduced non-uniformity between pixels and that between channels are effectively suppressed when CCD220 works in the multiplication CCD mode. The typical index for evaluating image non-uniformity is the photo response non-uniformity, i.e., PRNU, of which the original output image with poor uniformity is 24.5% when the gain voltage $\Delta v$ is 40.0 V, however, the uniformity of the image is significantly improved after the comprehensive correction, with the PRNU dramatically reduces to 4.1%, which proves the effectiveness of the proposed method. This method can effectively improve the uniformity of EMCCD output image, and provide a technical basis for EMCCD better application in various disciplines.
